# Intratumoral CXCR5^+^CD8^+^T associates with favorable clinical outcomes and immunogenic contexture in gastric cancer

**DOI:** 10.1038/s41467-021-23356-w

**Published:** 2021-05-25

**Authors:** Jieti Wang, Ruochen Li, Yifan Cao, Yun Gu, Hanji Fang, Yuchao Fei, Kunpeng Lv, Xudong He, Chao Lin, Hao Liu, Heng Zhang, He Li, Hongyong He, Jiejie Xu, Hua Huang

**Affiliations:** 1 Department of Gastric Surgery, Shanghai Cancer Center, Fudan University, Shanghai, China; 2 Department of Oncology, Shanghai Medical College, Fudan University, Shanghai, China; 3 Department of Surgical Oncology, Minhang Branch, Shanghai Cancer Center, Fudan University, Shanghai, China; 4 Department of General Surgery, Suzhou Science&Technology Town Hospital, Nanjing Medical University, Suzhou, China; 5 Department of General Surgery, Zhongshan Hospital, Fudan University, Shanghai, China; 6 Department of Biochemistry and Molecular Biology, School of Basic Medical Sciences, Fudan University, Shanghai, China

**Keywords:** Gastric cancer, Tumour biomarkers, Tumour immunology

## Abstract

Studies that examined an association between CD8^+^T and prognosis in gastric cancer are inconsistent, and a distinct population of CXCR5^+^CD8^+^T associated with better overall survival has been reported among various malignancies. Here, we show that the abundance of intratumoral CXCR5^+^CD8^+^T cells is associated with better overall survival in patients with gastric cancer. Patients with TNM II + III gastric cancer with higher intratumoral CXCR5^+^CD8^+^T cell infiltration are more likely to benefit from adjuvant chemotherapy. Microsatellite-unstable and Epstein–Barr virus positive tumors are enriched with CXCR5^+^CD8^+^T cells. Gastric cancer infiltrating CXCR5^+^CD8^+^T cells represent a specific subtype of stem-like CD8^+^T with effector memory feature. Identification of the clinical significance and phenotype of gastric cancer infiltrating CXCR5^+^CD8^+^T provides a roadmap for patient stratification and trials of targeted therapies.

## Abstract (web summary)

The prognostic value of tumor infiltrating lymphocytes in gastric cancer remains controversial. Here the authors show a consistent association between higher density of intratumoral CXCR5+CD8+ T cells and longer overall survival in four different cohorts of patients with gastric cancer.

## Introduction

In gastric cancer (GC), several attempts have been taken to associate the density of CD8^+^T with prognosis and therapeutic outcomes. Although CD8^+^T cells are believed to constitute the anti-tumor arm of tumor microenvironment, researches that examined an association between intratumoral CD8^+^T and prognosis in GC yielded inconsistent results^1–3^. A previous study^2^ showed an association of CD8^+^T and compromised overall survival (OS), which was contrary to the results of the later study^3^. These findings indicate the controversial prognostic effect and heterogeneous characteristics of CD8^+^T cells^4,5^ in GC.

Several studies have identified a subset of CD8^+^T that is characterized by the expression of CXCR5 in chronic lymphocytic choriomeningitis virus infection, which serves as a self-renewing exhausted progenitor and provides proliferation burst after programmed cell death protein 1 (PD-1) blockade^6–8^. Further studies have reported that the frequent density of CXCR5^+^CD8^+^T is associated with better OS in patients with lymphoma^9^, liver^10^, pancreas^11^, lung^12^, colorectum^13^, and thyroid^14^ malignancies. However, the prognostic value and the potential therapeutic benefits of targeting CXCR5^+^CD8^+^T in GC have not been explored.

Here we show that the abundance of intratumoral CXCR5^+^CD8^+^T is associated with better OS in patients with GC in four cohorts. And patients with tumor, node, metastasis (TNM) II + III disease whose tumor with higher CXCR5^+^CD8^+^T cell infiltration can benefit more from adjuvant chemotherapy (ACT). Furthermore, GC infiltrating CXCR5^+^CD8^+^T with stem-like effector memory CD8^+^T feature is enriched in microsatellite-unstable (MSI) and Epstein–Barr virus (EBV)-positive subsets of The Cancer Genome Atlas (TCGA) classification and MSI subset of Asian Cancer Research Group (ACRG) classification^15,16^.

## Results

### The presence of CXCR5^+^CD8^+^T cells in GC

**Fig. 1:**
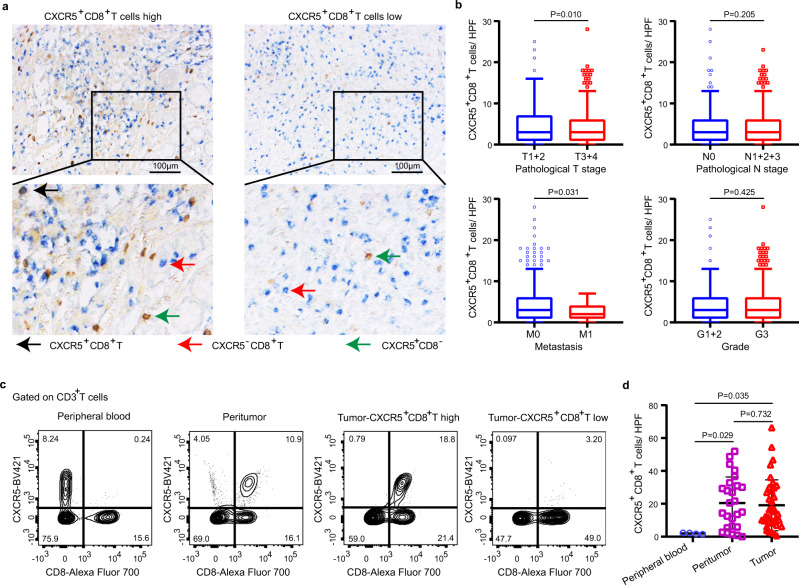
The presence of CXCR5^+^CD8^+^T cells in GC. **a** Representative IHC images of high and low CXCR5^+^CD8^+^T cell infiltration in GC tissue. Representative staining out of three independent experiments is shown. **b** Association of CXCR5^+^CD8^+^T with pathological T stage, pathological N stage, metastasis, and grade in ZSHS and FUSCC combined cohorts. Graph shows minima, maxima, center, bounds of box and whiskers, and percentile of *n* = 781 patients with gastric cancer for pathological T stage (*n* = 162 and *n* = 619 patients for T1 + 2 and T3 + 4 subgroups, respectively), pathological N stage (*n* = 242 and *n* = 539 patients for N0 and N1 + 2 + 3 subgroups, respectively), metastasis (*n* = 758 and *n* = 23 patients for M0 and M1 subgroups, respectively), and grade (*n* = 192 and *n* = 589 patients for G1 + 2 and G3 subgroups, respectively). Statistical analysis by two-tailed unpaired *t* test. **c** Representative flow cytometric images of CXCR5^+^CD8^+^T cells in peripheral blood, peritumor, and tumor tissues. **d** The presence of CXCR5^+^CD8^+^T cells in peripheral blood, peritumor, and tumor tissues. Graph shows mean ± SD of *n* = 4 (peripheral blood), *n* = 26 (peritumor tissues), and *n* = 37 (tumor tissues) individual patients. Statistical analysis by two-tailed unpaired *t* test. Source data are provided as a Source data file. HPF high-power field.

To identify the presence of CXCR5^+^CD8^+^T cells in GC, we performed double immunohistochemistry (IHC) staining and flow cytometry (Fig. 1). As shown, there are more CXCR5^+^CD8^+^T cells in lower pathological T stage and non-metastatic tumors (Fig. 1a, b). And further analysis validates that CXCR5^+^CD8^+^T cells are mainly present in tumor and peritumor tissues, and only 3% of CD8^+^T cells presented in peripheral blood express CXCR5 (Fig. 1c, d).

The CXCR5^+^CD8^+^T population has been reported to be implicated in a niche reminiscent of tertiary lymphoid structure (TLS) in the tumor tissues^17^. To investigate whether this subset resides in a TLS-like niche, we further performed multiple immunofluorescence (IF) and hematoxylin–eosin staining in continuous slides. As shown in Supplementary Fig. 1, CXCR5^+^CD8^+^T cells dominantly reside in the TLS-like niche, whereas there are scattered CXCR5^+^CD8^+^T cells residing in the non-TLS area.

### CXCR5^+^CD8^+^T acts as a better prognosticator than CD8^+^T in GC

**Fig. 2:**
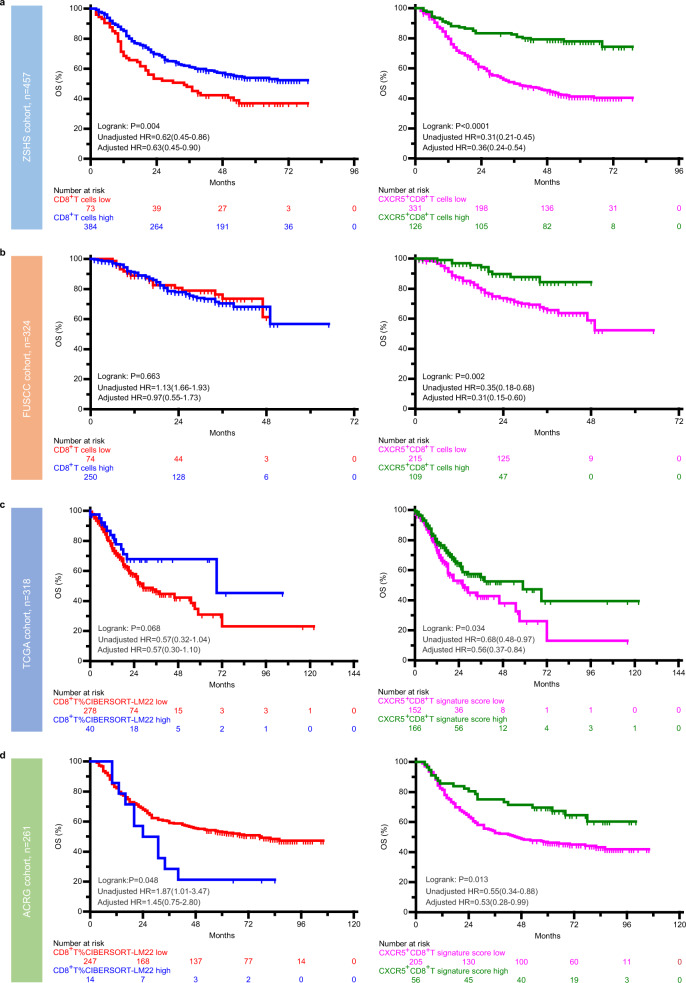
Intratumoral CXCR5^+^CD8^+^T is associated with better OS. **a**–**d** Survival curves for CD8^+^T/CXCR5^+^CD8^+^T cell infiltration (high and low) for OS in the **a** ZSHS, **b** FUSCC, **c** TCGA, and **d** ACRG cohorts. *P* value shown resulted from the comparison between CD8^+^T/CXCR5^+^CD8^+^T cell low and high groups of *n* = 457 (ZSHS cohort), *n* = 324 (FUSCC cohort), *n* = 318 (TCGA cohort), and *n* = 261 (ACRG cohort) patients with gastric cancer, using the Kaplan–Meier method followed by log-rank statistical test; the unadjusted and adjusted HRs and 95% CIs are reported. Source data are provided as a Source data file. ZSHS Zhongshan Hospital, FUSCC Fudan University Shanghai Cancer Center, TCGA The Cancer Genome Atlas, ACRG Asian Cancer Research Group, HR hazard ratio.

To compare the prognostic value of intratumoral CD8^+^T and CXCR5^+^CD8^+^T in patients with GC, we conducted Kaplan–Meier curves in Zhongshan Hospital cohort (ZSHS, *n* = 457), Fudan University Shanghai Cancer Center cohort (FUSCC, *n* = 324), TCGA cohort (*n* = 318), and ACRG cohort (*n* = 261). The patient characteristics in four cohorts are presented in Supplementary Tables 1 and 2 and the flowchart of patients enrolled and study design are presented in Supplementary Fig. 2. The association between CD8^+^T and prognosis is inconsistent in the four cohorts, with favorable clinical implication of the presence of CD8^+^T in the ZSHS cohort (*P* = 0.004; Fig. 2a), without statistical significance in the FUSCC and TCGA cohorts (*P* = 0.663 and *P* = 0.068, respectively; Fig. 2b, c), and shorter OS duration in the ACRG cohort (*P* = 0.048; Fig. 2d). However, the association between CXCR5^+^CD8^+^T and prognosis remains consistent, with CXCR5^+^CD8^+^T-high group having longer OS than the CXCR5^+^CD8^+^T-low group in the four cohorts (*P* < 0.001, *P* = 0.002, *P* = 0.034, and *P* = 0.013, respectively; Fig. 2). The abundance of CXCR5^+^CD8^+^T remains to be a favorable prognosticator after adjustment for age, gender, histotype, grade, pathological T stage, pathological N stage, metastasis, MSI status, and EBV as confounders in four cohorts, while CD8^+^T cell infiltration fails to be statistical significance after adjustment for confounders in the FUSCC, TCGA, and ACRG cohorts (Fig. 2 and Supplementary Fig. 3).

### Association of CXCR5^+^CD8^+^T with response to ACT

**Fig. 3:**
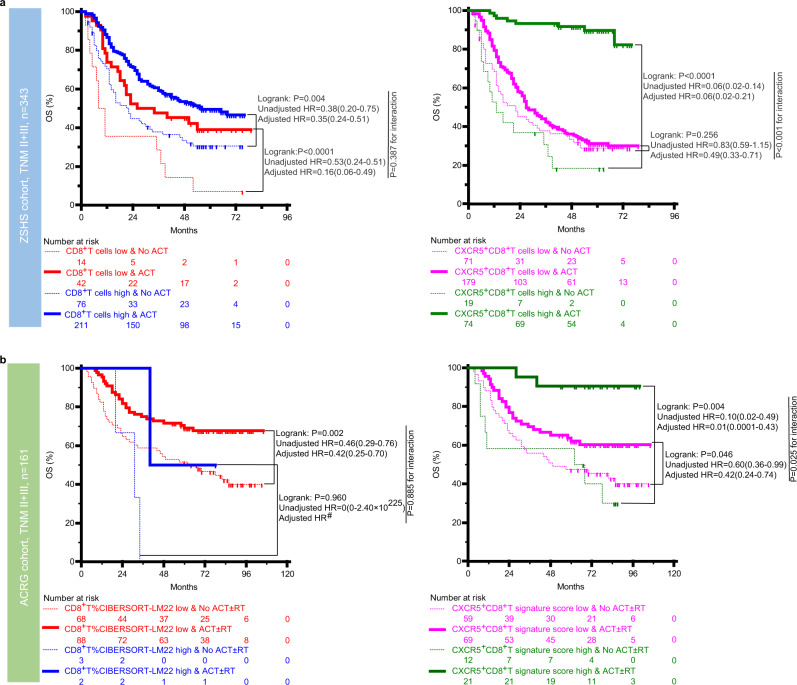
CXCR5^+^CD8^+^T is associated with superior response to ACT. **a**, **b** Survival curves for the use of ACT (±RT) in patients with high/low CD8^+^T/CXCR5^+^CD8^+^T cell infiltration in the **a** ZSHS and **b** ACRG cohorts. HRs of disease mortality for patients with ACT (±RT) versus without according to tumor CD8^+^T/CXCR5^+^CD8^+^T cell infiltration and interaction between the biomarker and treatment were reported. The response to ACT (±RT) analysis was conducted in patients with TNM II + III stage diseases. *P* value shown resulted from the comparison between with and without ACT (±RT) treatment of *n* = 343 (ZSHS TNM II + III cohort) and *n* = 161 (ACRG TNM II + III cohort) patients with gastric cancer, using the Kaplan–Meier method followed by log-rank statistical test; the unadjusted and adjusted HRs and 95% CIs are reported. ^#^Sample size too small to conduct multivariate Cox analysis. Source data are provided as a Source data file. ZSHS Zhongshan Hospital, ACRG Asian Cancer Research Group, HR hazard ratio, CI confidence interval, ACT adjuvant chemotherapy, RT radiotherapy.

The association between ACT (±radiotherapy (RT)) and better OS in patients with TNM II + III GC has been identified by the MAGIC^18^, CLASSIC^19^, and CRITICS^20^ trials. The patient characteristics in ZSHS and ACRG TNM II + III cohorts are presented in Supplementary Table 3. To evaluate whether patients with CXCR5^+^CD8^+^T-high tumor might benefit more from ACT (±RT) compared with patients with CXCR5^+^CD8^+^T-low tumor, we investigated the association between CXCR5^+^CD8^+^T cells density and OS among patients with TNM II + III diseases who either did or did not receive ACT. The results confirm that patients treated with ACT have a higher rate of OS in the CXCR5^+^CD8^+^T-high patient population of the ZSHS (*P* < 0.001; Fig. 3a) and ACRG (*P* < 0.001; Fig. 3b) cohorts. A test for the interaction between the biomarker and the treatment reveals that the benefit observed in CXCR5^+^CD8^+^T-high subgroups is superior to that observed in CXCR5^+^CD8^+^T-low subsets in both the ZSHS (*P* < 0.001) and ACRG (*P* = 0.025) cohorts. However, the test for the interaction between the density of CD8^+^T cells and treatment fails to be statistically significant in both the ZSHS (*P* = 0.387) and ACRG (*P* = 0.885, the result should be treated cautiously due to the limited number of individuals) cohorts. Furthermore, multivariate Cox analyses reveal that patients with ACT have lower risk of mortality than patients without ACT after adjustment for confounders across the subgroups (Fig. 3 and Supplementary Tables 4 and 5).

### Association of CXCR5^+^CD8^+^T with TCGA/ACRG classification

**Fig. 4:**
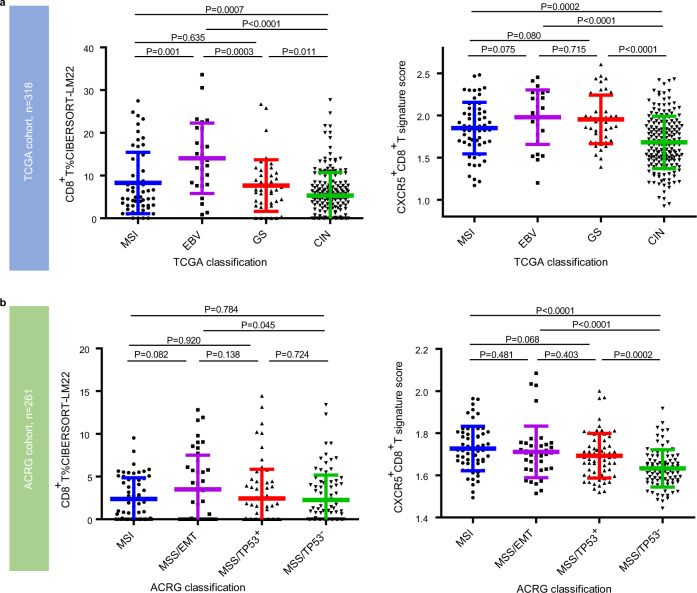
Association of CXCR5^+^CD8^+^T with TCGA/ACRG classification. **a** CD8^+^T%CIBERSORT-LM22 and CXCR5^+^CD8^+^T signature score in the MSI, EBV, GS, and CIN subgroups of the TCGA cohort (the TCGA classification, *n* = 318 patients with gastric cancer). Graph shows mean ± SD of *n* = 62 (MSI), *n* = 27 (EBV), *n* = 46 (GS), and *n* = 183 (CIN) individual patients, using statistical analysis by two-tailed unpaired *t* test. **b** CD8^+^T%CIBERSORT-LM22 and CXCR5^+^CD8^+^T signature score in the MSI, MSS/EMT, MSS/TP53^+^, and MSS/TP53^−^ subgroups of the ACRG cohort (the ACRG classification, *n* = 261 patients with gastric cancer). Graph shows mean ± SD of *n* = 61 (MSI), *n* = 43 (MSS/EMT), *n* = 68 (MSS/TP53^+^), and *n* = 89 (MSS/TP53^−^) individual patients, using statistical analysis by two-tailed unpaired *t* test. Source data are provided as a Source data file. TCGA The Cancer Genome Atlas, ACRG Asian Cancer Research Group, MSI micro-satellite unstable, EBV Epstein–Barr virus, GS genomically stable, CIN chromosomal instability, MSS micro-satellite stable, EMT mesenchymal-like type, TP53 tumor protein 53.

The molecular subtypes and their association with prognosis and patient therapeutic strategies have been established by the TCGA^15^ and ACRG^16^ research networks. To integrate tumor microenvironment characteristics and molecular subtypes, we sought to determine the association between CD8^+^T/CXCR5^+^CD8^+^T and TCGA/ACRG classifications. In the TCGA cohort, the density of CD8^+^T is high in the EBV subtype but is low in the MSI, genomically stable (GS), and chromosomal instability (CIN) subtypes. However, the CXCR5^+^CD8^+^T signature score is relatively high in the MSI, EBV, and GS subtypes but is the lowest in the CIN subtype (*P* < 0.001, *P* < 0.0001, and *P* < 0.0001, respectively; Fig. 4a). In the ACRG cohort, the presence of CD8^+^T is high in the microsatellite stable (MSS)/epithelial-to-mesenchymal transition (EMT) subtype but was low in the MSI, MSS/tumor protein 53-active (TP53^+^), and MSS/TP53-inactive (TP53^−^) subtypes. Interestingly, the CXCR5^+^CD8^+^T signature score is the highest in the MSI subtype, intermediate in the MSS/EMT and MSS/TP53^+^ subtypes, but the lowest in the MSS/TP53^−^ subtype (*P* < 0.0001, *P* < 0.0001, and *P* < 0.001, respectively; Fig. 4b).

To investigate whether the results in the overall population are driven by MSI and EBV subgroups that are enriched for CXCR5^+^CD8^+^T-high tumors, we merged the ZSHS, TCGA, and ACRG cohorts into a combined cohort (*n* = 1036) and conducted the subgroup analyses in the combined cohort. As shown in Supplementary Fig. 4, patients with CXCR5^+^CD8^+^T-high tumor have superior OS than patients with CXCR5^+^CD8^+^T-low tumor in the MSI-low/MSS, MSI-high, and EBV-negative subgroups (*P* < 0.001, *P* = 0.006, and *P* = 0.034, respectively; Supplementary Fig. 4a, b). Further univariate Cox analyses reveal that patients with MSI-low/MSS and CXCR5^+^CD8^+^T-low tumor or patients with EBV-negative and CXCR5^+^CD8^+^T-low tumor have worst outcome (Supplementary Fig. 4c, d).

### GC infiltrating CXCR5^+^CD8^+^T is a subset of stem-like CD8^+^T with effector memory characteristic

**Fig. 5:**
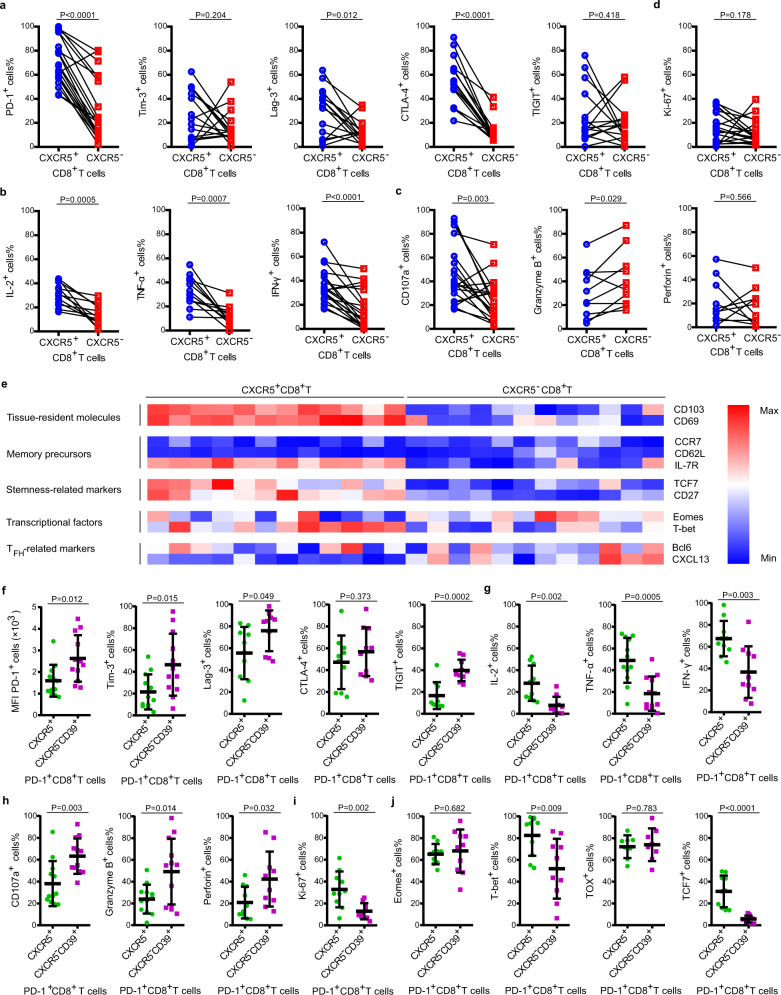
Phenotype of CXCR5^+^CD8^+^T cells presented in GC. **a** The immune-checkpoint of CXCR5^+^CD8^+^T cells and CXCR5^−^CD8^+^T cells from GC tissues. Graph shows mean ± SD of each with *n* = 20 (PD-1), *n* = 15 (Tim-3), *n* = 15 (Lag-3), *n* = 15(CTLA-4), and *n* = 15 (TIGIT) individual patients. **b** The cytokine of CXCR5^+^CD8^+^T cells and CXCR5^−^CD8^+^T cells from GC tissues. Graph shows mean ± SD of each with *n* = 12 (IL-2), *n* = 12 (TNF-α), and *n* = 20 (IFN-γ) individual patients. **c** The effecter molecules of CXCR5^+^CD8^+^T cells and CXCR5^−^CD8^+^T cells from GC tissues. Graph shows mean ± SD of each with *n* = 20 (CD107a), *n* = 10 (granzyme B), and *n* = 10 (perforin) individual patients. **d** The proliferative potential of CXCR5^+^CD8^+^T cells and CXCR5^−^CD8^+^T cells from GC tissues. Graph shows mean ± SD of each with *n* = 20 (Ki-67) individual patients. **a**–**d** Statistical analysis by two-tailed paired *t* test. **e** Heat map illustrating the relative expression of molecules in CXCR5^+^CD8^+^T cells and CXCR5^−^CD8^+^T cells from *n* = 12 patients with gastric cancer. **f** The exhaustion markers of PD-1^+^CXCR5^+^CD8^+^T cells and PD1^+^CD39^+^CXCR5^−^CD8^+^T cells from GC tissues. Graph shows mean ± SD of each with *n* = 12 (PD-1), *n* = 12 (Tim-3), *n* = 10 (Lag-3), *n* = 10 (CTLA-4), and *n* = 10 (TIGIT) individual patients. **g** The cytokine of PD-1^+^CXCR5^+^CD8^+^T cells and PD1^+^CD39^+^CXCR5^−^CD8^+^T cells from GC tissues. Graph shows mean ± SD of each with *n* = 10 (IL-2), *n* = 12 (TNF-α), and *n* = 10 (IFN-γ) individual patients. **h** The effecter molecules of PD-1^+^CXCR5^+^CD8^+^T cells and PD1^+^CD39^+^CXCR5^−^CD8^+^T cells from GC tissues. Graph shows mean ± SD of each with *n* = 12 (CD107a), *n* = 12 (granzyme B), and *n* = 10 (perforin) individual patients. **i** The proliferative potential of PD-1^+^CXCR5^+^CD8^+^T cells and PD1^+^CD39^+^CXCR5^−^CD8^+^T cells from GC tissues. Graph shows mean ± SD of each with *n* = 10 (Ki-67) individual patients. **j** The transcription factors of PD-1^+^CXCR5^+^CD8^+^T cells and PD1^+^CD39^+^CXCR5^−^CD8^+^T cells from GC tissues. Graph shows mean ± SD of each with *n* = 10 (Eomes), *n* = 10 (T-bet), *n* = 8 (TOX), and *n* = 10 (TCF7) individual patients. **f**–**j** Statistical analysis by two-tailed unpaired *t* test. Source data are provided as a Source data file.

To explore the mechanism underlined the preceding observations, we therefore investigated the features of CXCR5^+^CD8^+^T presented in tumor tissues by using the fresh surgical specimens of GC. Notably, CXCR5^+^CD8^+^T cells in GC express higher levels of immune-checkpoint molecules^4,21^ including PD-1, Lag-3, and CTLA-4, compared with their CXCR5^−^ counterparts (Fig. 5a). Furthermore, CXCR5^+^CD8^+^T cells have a higher level of cytokines (interleukin (IL)-2, tumor necrosis factor (TNF)-α, and interferon (IFN)-γ) and CD107a but a lower level of granzyme B (Fig. 5b, c), suggesting a less terminated differentiated state^22,23^. Interestingly, the two CD8^+^T subsets express comparable Ki-67, but CXCR5^+^CD8^+^T cells have higher tissue-resident^24–26^ (CD103 and CD69) and stemness-related marker^27–29^ (TCF7 and CD27) expression (Fig. 5d, e). Regarding transcription factors, both subsets express Eomes and T-bet^30,31^, CXCR5^+^CD8^+^T cells express higher T-bet and lower Eomes, while CXCR5^−^CD8^+^T cells show higher Eomes and lower T-bet expression. The expression of central memory cell markers^32^ (CCR7 and CD62L) is low in both subsets, while the effector memory cell marker^32^ IL-7R is high in CXCR5^+^CD8^+^T cells. Surprisingly, some CXCR5^−^CD8^+^T cells show Bcl-6 expression, and CXCL-13 is highly expressed by a part of CXCR5^−^CD8^+^T cells (Fig. 5e), which may suggest the exhausted phenotype of CXCR5^−^CD8^+^T cells^33,34^.

It has been reported that a majority of CD8^+^T cells are bystander cells^35^. However, CXCR5^+^CD8^+^T cells largely represent tumor-specific CD8^+^T cells. Therefore, we further compared PD-1^+^CXCR5^+^CD8^+^T cells with PD1^+^CD39^+^CXCR5^−^CD8^+^T cells to explore the features of tumor-specific CD8^+^T cells. Interestingly, PD-1^+^CXCR5^+^CD8^+^T cells in GC express lower levels of exhaustion markers (PD-1, Tim-3, Lag-3, and TIGIT; Fig. 5f) and higher level of cytokines (IL-2, TNF-α, and IFN-γ; Fig. 5g), but a lower level of effector molecules (granzyme B and perforin, Fig. 5h) compared with PD1^+^CD39^+^CXCR5^−^CD8^+^T cells. Furthermore, the PD-1^+^CXCR5^+^CD8^+^T subset has a higher Ki-67 expression (Fig. 5i). Regarding transcription factors, the two tumor-specific CD8^+^T subsets expressed comparable Eomes and TOX, while PD-1^+^CXCR5^+^CD8^+^T cells show higher T-bet and TCF7 expression (Fig. 5j).

## Discussion

As expected, our study identifies that the association between CD8^+^T and OS is inconsistent. However, we found a distinct population of CXCR5^+^CD8^+^T presented in GC and an association between higher density of CXCR5^+^CD8^+^T and longer OS duration by using four patient cohorts. Given the size of the data sets and the confounders considered for adjustment, this study provides a reliable evidence for the independently prognostic role of CXCR5^+^CD8^+^T.

Remarkably, the current study reveals the predictive value of CXCR5^+^CD8^+^T in response to ACT (±RT). Patients with TNM II + III disease whose tumor with higher CXCR5^+^CD8^+^T have superior OS benefit from ACT (±RT). We note, however, a different risk reduction in the ZSHS cohort compared with the ACRG cohort, even after adjustment for interaction and confounders, which might be explained by the different enrolled populations and the administration of RT to a part of patients in the ACRG cohort. Meanwhile, there are likely drivers of eligibility for treatment that impact prognosis and are hard to adjust for, and further prospective study validation needs to be conducted.

Furthermore, it has been reported that patients with the MSI-high and EBV-positive GC dramatically responded to anti-PD-1 therapy^36^. We have shown that CXCR5^+^CD8^+^T cells with high PD-1 expression intensely presented in the TCGA classification the MSI and EBV subtypes, and in the ACRG classification MSI subtype of GC, which might partially explain the underlining mechanism of superior response to anti-PD-1 therapy in MSI-high and EBV-positive GC. Another observation is the presence of CXCR5^+^CD8^+^T cells in the GS subtype of the TCGA classification, MSS/EMT and MSS/TP53^+^ subtypes of the ACRG subtypes, and considering the expression pattern of PD-1/CTLA-4 in CXCR5^+^CD8^+^T cells, these results suggest that these subtypes of GC might response to anti-PD-1/anti-CTLA-4 combined therapy.

In summary (Supplementary Fig. 5), the abundance of intratumoral CXCR5^+^CD8^+^T is associated with better OS and patients with TNM II + III disease whose tumor presented higher intratumoral CXCR5^+^CD8^+^T cells could benefit more from ACT. This stem-like CXCR5^+^CD8^+^T with effector memory feature is highly presented in the TCGA classification MSI, EBV, and GS subtypes and the ACRG classification MSI, MSS/EMT, and MSS/TP53^+^ subtypes of GC. Given the superior prognostic value and predictive value of response to ACT of CXCR5^+^CD8^+^T in GC compared with CD8^+^T, CXCR5^+^CD8^+^T might be used as a biomarker and a therapeutic target in GC.

## Methods

### Study design and patients

The study included four patient cohorts, ZSHS (*n* = 457) cohort, FUSCC (*n* = 324) cohort, TCGA cohort (*n* = 318), and ACRG cohort (*n* = 261). The ZSHS and FUSCC cohorts consist of patients with GC from ZSHS (Shanghai, China) and FUSCC (Shanghai, China) who received gastrectomy during December 2007―December 2008 and January 2010―December 2011, respectively. All data were gathered retrospectively, and the survival periods were defined as months after surgery. Patients with ACT were defined as patients who received at least one cycle of 5-fluorouracil-based ACT (median: 6 cycles; range: 1―8 cycles). Patient characteristics of the TCGA^15^ and ACRG^16^ cohorts were retrieved from http://www.cbioportal.org/study/summary?id=stad_tcga on 4 June 2018 and https://www.ncbi.nlm.nih.gov/geo/query/acc.cgi?acc=GSE62254 on 15 January 2019, respectively. Peripheral blood and fresh tissue specimens were collected from treated naive and surgically resectable patients (*n* = 63) with GC who received gastrectomy during September 2018―October 2019 and August 2020―September 2020 at FUSCC and October 2020 at ZSHS. Informed consents from patients and research protocol were approved by the Clinical Research Ethics Committee of Fudan University Zhongshan Hospital and the Clinical Research Ethics Committee of Fudan University Shanghai Cancer Center.

### Multiplex IHC and IF

Formalin-fixed paraffin-embedded surgical specimens of GC were collected and sectioned for slides. The slides were dewaxed for 6 h at 65 °C in a dry-heat oven, deparaffinized in xylene for 15 min, washed with 100% ethanol followed by 95, 85, and 75% ethanol, and then rinsed in Tris-buffered saline with Tween-20 (TBST) for 3 times. For antigen retrieval, slides were boiled for 14 min in 10 mM sodium citrate buffer (pH 6.0). After 3 washes with TBST, slides were treated for 30 min at 37 °C in 3% H_2_O_2_ and then rinsed in TBST for 3 times. Slides were blocked for 2 h at 37 °C in 10% goat serum blocking buffer, and then incubated overnight at 4 °C with primary antibodies diluted in blocking buffer. The detailed information of IHC and IF antibodies is described in Supplementary Table 6.

For IHC, the EnVision System HRP/Rabbit and AP/Mouse were applied (Dako) with 3,30-diaminobenzidine and Vector Blue to visualize the reaction products, respectively. And for IF, Alexa Flour 647 Goat Anti-Mouse IgG (Abcam) and Alexa Flour 488 Goat Anti-Rabbit IgG (Abcam) were used for visualization. Digital images of IHC and IF were taken using Image Pro plus 6.0 (Media Cybernetics Inc.) and 3DHISTECH’s Viewer Application (3DHISTECH Ltd.) under high-power magnification filed (HPF, ×200 magnification), respectively. The density of CXCR5^+^CD8^+^T, CXCR5^−^CD8^+^T, and CXCR5^+^CD8^−^ was recorded as the mean number of cells/HPF from 6 randomized fields counted by 2 independent pathologists (each with 3 fields) who were blinded from the clinical data.

### Flow cytometry

Freshly collected peripheral blood and excised specimens were stored at 4 °C and then used for analysis within 4 h. Peripheral blood mononuclear cells were isolated by Ficoll-Hypaque through density gradient centrifugation. To obtain single-cell suspensions, we minced fresh excised specimens, digested them with 2 mg/ml type IV collagenase (Sigma) and 50 U/ml DNAse (Sigma) in RPMI 1640 for 1.5 h at 37 °C, and then filtered through a 70-μm nylon mesh.

For CXCR5^+^CD8^+^T cell quantification, preceding collected cells were washed once in cold phosphate-buffered saline, and stained in Stain Buffer (BD Bioscience) with a panel of fluorochrome-tagged monoclonal antibodies (Supplementary Table 6). Red cells were lysed with an ammonium chloride solution and samples were incubated with 7-aminoactinomycin D for 5 min and washed prior to staining to permit identification of live cells. Samples stained with isotype-matched antibodies were used as negative controls. For sample acquisition, a BD FACSCanto II flow cytometer with FACS DIVA software was used (BD Bioscience) and FlowJo vX.0.7 software was used for the analyses. The gating strategy for CD3^+^T is presented in Supplementary Fig. 6.

### Bioinformatics

To analyze the density of CD8^+^T cells in the TCGA and ACRG cohorts, we used the CIBERSORT platform^37^ (Stanford University, CA, USA, https://cibersort.stanford.edu/) to calculate the relative proportion of CD8^+^T cells in various immune cell types recognized as CIBERSORT-LM22. To analyze the density of CXCR5^+^CD8^+^T cells, we used the average mean of the mRNA expression of *CD8A*, *CXCR5*, *CXCR3*, *ICOS*, *CD27*, *IL21*, *TNF*, *TNFRSF6B*, *PDCD1*, *TBX21*, *SLAMF6*, and *IL27RA* to constitute the CXCR5^+^CD8^+^T signature score^38,39^. In order to control the range, we pre-calculated the mRNA expression in the TCGA cohort with lg (*x* + 1).

The cutoff points were determined automatically by the Cutoff Finder platform^40^ (Charité–Universitätsmedizin Berlin, Berlin, Germany, http://molpath.charite.de/cutoff). The cutoff points of CD8^+^T cells are 36 cells/HPF for the ZSHS and FUSCC cohorts. As for TCGA and ACRG cohorts with different RNA-seq normalization method, the cutoff points of CD8^+^T%CIBERSORT-LM22 are 14.022 and 8.890%, respectively. The cutoff points of CXCR5^+^CD8^+^T cells are 6 cells/HPF for the ZSHS and FUSCC cohorts. As for TCGA and ACRG cohorts, the cutoff points of CXCR5^+^CD8^+^T signature score are 1.770 and 1.760.

### Statistical analysis

In individual cohort analysis, the association between CXCR5^+^CD8^+^T and clinical factors were evaluated by Student’s *t* test (continuous variables), Chi-squared test (categorical variables), and Fisher’s exact test (categorical variables violating the rules of Chi-squared test). Kaplan–Meier curves and logrank (Mantel–Cox) tests were conducted for survival analysis. Univariate and multivariate analyses were performed by Cox proportional-hazards regression, and hazard ratio and 95% confidence intervals were reported. Interactions between the biomarker and ACT were evaluated with the Cox proportional-hazards method. Unpaired Student’s t test was used to detect statistically significant differences between the TCGA/ACRG classification subgroups. For fresh specimen analysis, all results are presented as the mean ± standard deviation (SD). Two-sided *P* < 0.05 was considered statistically significant. Medcalc v12.7.0, SPSS v21.0, and GraphPad Prism v8.0.1 were used for statistical analyses. Heat map was conducted by Funrich v3.1.3.

### Reporting summary

Further information on research design is available in the Nature Research Reporting Summary linked to this article.

## Supplementary information

Supplementary Information

Reporting Summary

## Data Availability

Patient characteristics of the TCGA and ACRG cohorts are publicly available in the TCGA database [http://www.cbioportal.org/study/summary?id=stad_tcga] and in GEO database under accession code GSE62254, respectively. The remaining data supporting the findings of this study are available within the article and its supplementary information files or available from the authors upon request. Source data are provided with this paper.

## Source data

Source Data
