# Supplementary material for: Intratumoral CXCR5+CD8+T associates with favorable clinical outcomes and immunogenic contexture in gastric cancer
Source: Nat Commun. 2021 May 25;12:3080. doi: 10.1038/s41467-021-23356-w (PMC8149695; doi:10.1038/s41467-021-23356-w)
Supplement: Supplementary file 1 — Supplementary Information [file 41467_2021_23356_MOESM1_ESM.pdf]

SUPPLEMENTARY INFORMATION

Intratumoral $CXCR5^+CD8^+T$ associates with favorable clinical outcomes and immunogenic contexture in gastric cancer

Wang et al.

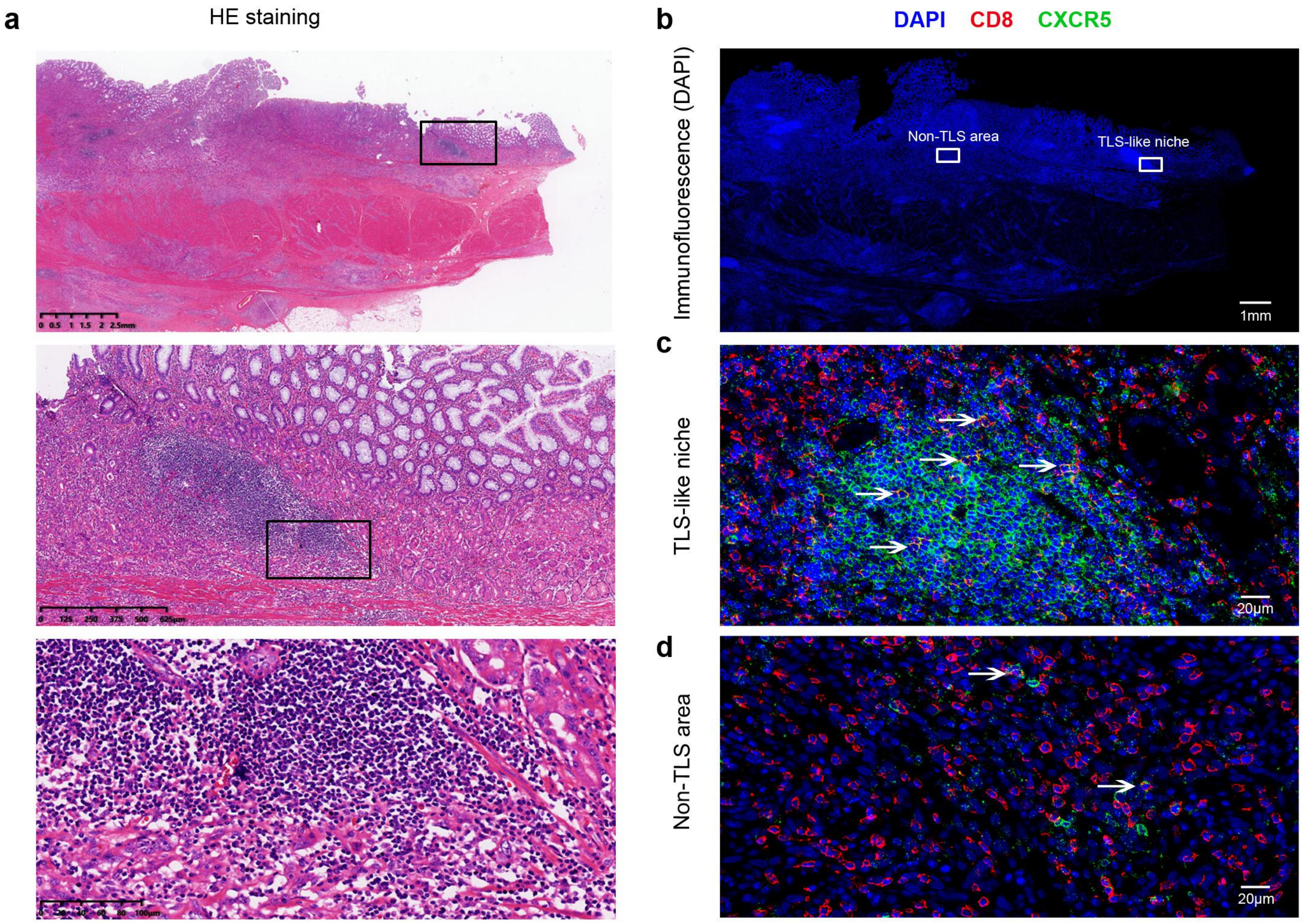

Supplementary Fig. 1 The location of CXCR5$^+$CD8$^+$T cells in gastric cancer. a Representative HE staining of TLS in GC tissue. **b** Representative immunoflourescence DAPI of continuous GC tissue slide. **c**, **d** CXCR5$^+$CD8$^+$T cells in the TLS-like niche (**c**) and non-TLS area (**d**) of GC tissue. TLS means tertiary lymphoid structure. Representative staining out of three independent experiments shown.

**Step 1. Enrolled patient cohorts**	Zhongshan Hospital cohort (raw data, *n* = 482)<br>*n* = 13 without intact clinical information<br>*n* =12 with missing specimens<br>**ZSHS cohort, *n* = 457**	Fudan University Shanghai Cancer Center cohort (raw data, *n* = 352)<br>*n* = 27 without intact clinical information<br>*n* =1 with missing specimens<br>**FUSCC cohort, *n* = 324**	The Cancer Genome Altlas cohort (raw data, *n* = 406)<br>*n* = 88 without intact clinical information<br>**TCGA cohort, *n* = 318**	Asian Cancer Research Group cohort (raw data, *n* = 300)<br>*n* = 39 without intact clinical information<br>**ACRG cohort, *n* = 261**
**Step 2. Immunocytes infiltration evaluation**	Multiplex IHC staining<br>Pathological evaluation<br>$CXCR5^+CD8^+T$ cells/HPF<br>$CD8^+T$ cells/HPF	Multiplex IHC staining<br>Pathological evaluation<br>$CXCR5^+CD8^+T$ cells/HPF<br>$CD8^+T$ cells/HPF	CIBERSORT ananlysis<br>$CD8^+T$%CIBERSORT-LM22<br>$CXCR5^+CD8^+T$ signature score	CIBERSORT analysis<br>$CD8^+T$%CIBERSORT-LM22<br>$CXCR5^+CD8^+T$ signature score
**Step 3. Survival analysis**	Find the cutoff points<br>Association with OS<br>Univariate/multivariate cox analysis	Find the cutoff points<br>Association with OS<br>Univariate/multivariate cox analysis	Find the cutoff points<br>Association with OS<br>Univariate/multivariate cox analysis	Find the cutoff points<br>Association with OS<br>Univariate/multivariate cox analysis
**Step 4. Adjuvant chemotherapy response analysis**	**TNM II+III** were included, *n* = 343<br>Association with response to ACT<br>HR (95%CI) analysis	**Skiped** (without data)	**Skiped** (without data)	**TNM II+III** were included, *n* = 161<br>Association with response to ACT ± RT<br>HR (95%CI) analysis
**Step 5. TCGA/ACRG classification analysis**	**Skiped** (without data)	**Skiped** (without data)	Association with TCGA classification	Association with ACRG classification

Supplementary Fig. 2 Flowchart for study design. The enrolled criterion and conducted analyses in the ZSHS (*n* = 457 individual patients), FUSCC (*n* = 324 individual patients), TCGA (*n* = 318 individual patients), and ACRG (*n* = 261 individual patients) cohorts.

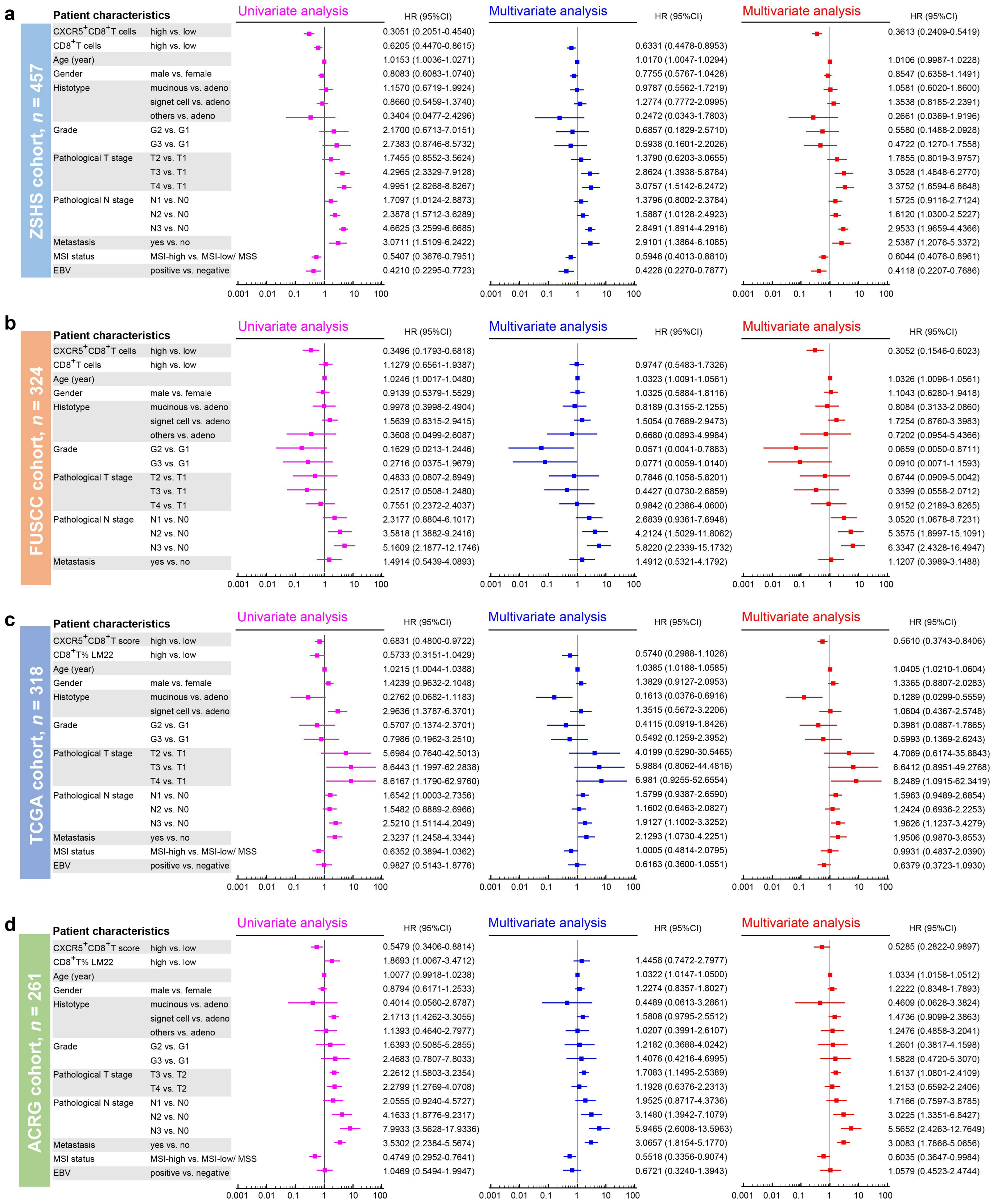

Supplementary Fig. 3 Univariate and multivariate Cox analyses. a-d Univariate and multivariate analyses for HRs with 95%CIs of patient characteristics in the (**a**) ZSHS (*n* = 457 individual patients), (**b**) FUSCC (*n* = 324 individual patients), (**c**) TCGA (*n* = 318 individual patients), and (**d**) ACRG (*n* = 261 individual patients) cohorts. Univariate and multivariate analyses were performed by Cox proportional-hazards regression, and HRs and 95%CIs were reported.

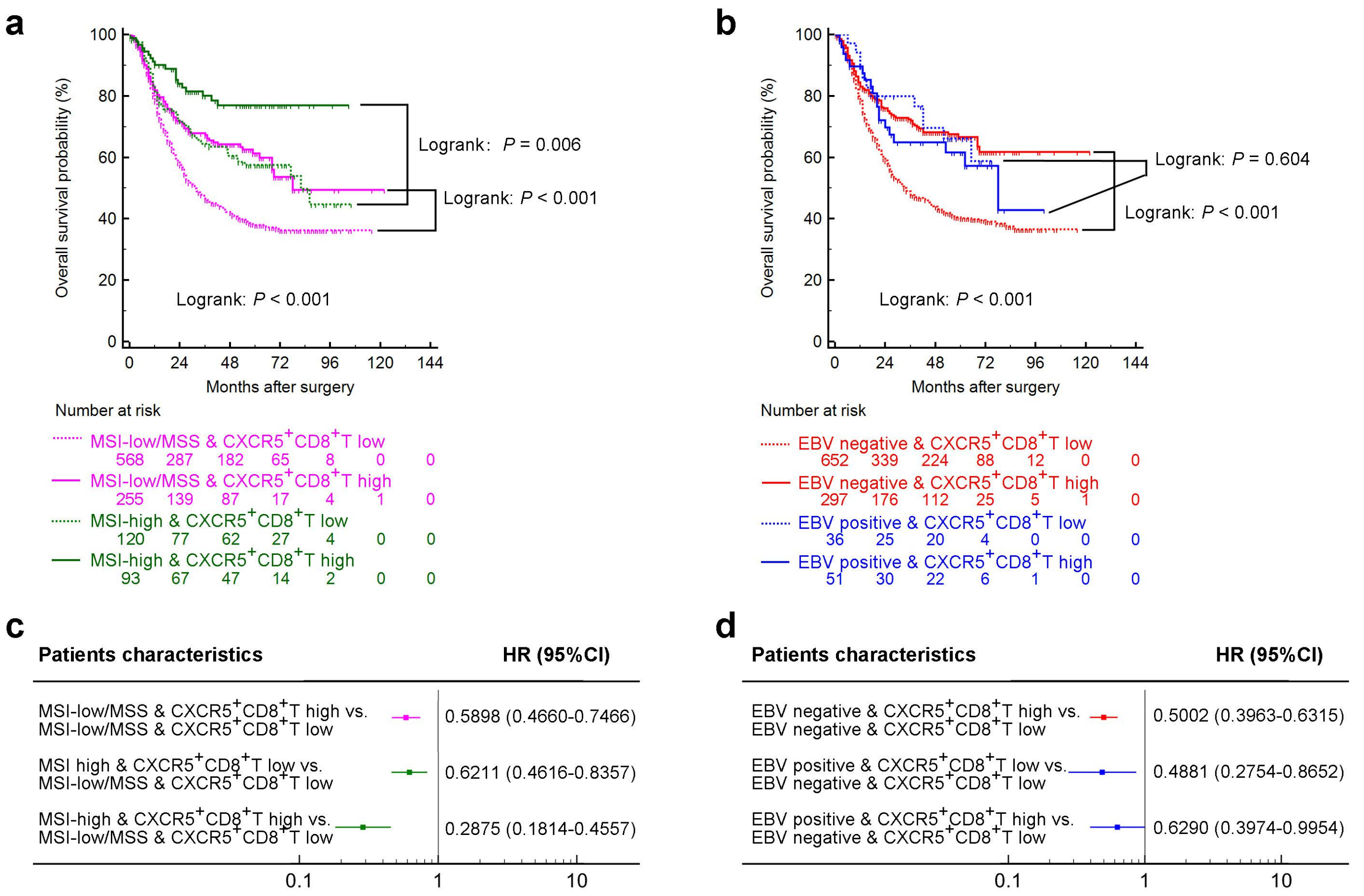

Supplementary Fig. 4 Subgroup analyses in the ZSHS, TCGA, and ACRG combined cohort. a Survival curves for CXCR5^{+}CD8^{+}T (high and low) for OS in the MSI-low/MSS subgroup with n = 823 patients and MSI-high subgroup with n = 213 patients. P value indicates the Kaplan-Meier method followed by log-rank statistical test. **b** Survival curves for CXCR5^{+}CD8^{+}T (high and low) for OS in the EBV negative subgroup with n = 949 patients and EBV positive subgroup with n = 87 patients. P value indicates the Kaplan-Meier method followed by log-rank statistical test. **c**, **d** Univariate Cox analyses for HRs with 95%CIs of patient characteristics in the combined cohort with n = 1036 patients. Univariate analyses were performed by Cox proportional-hazards regression, and HRs and 95%CIs were reported. Source data are provided as a Source Data file.

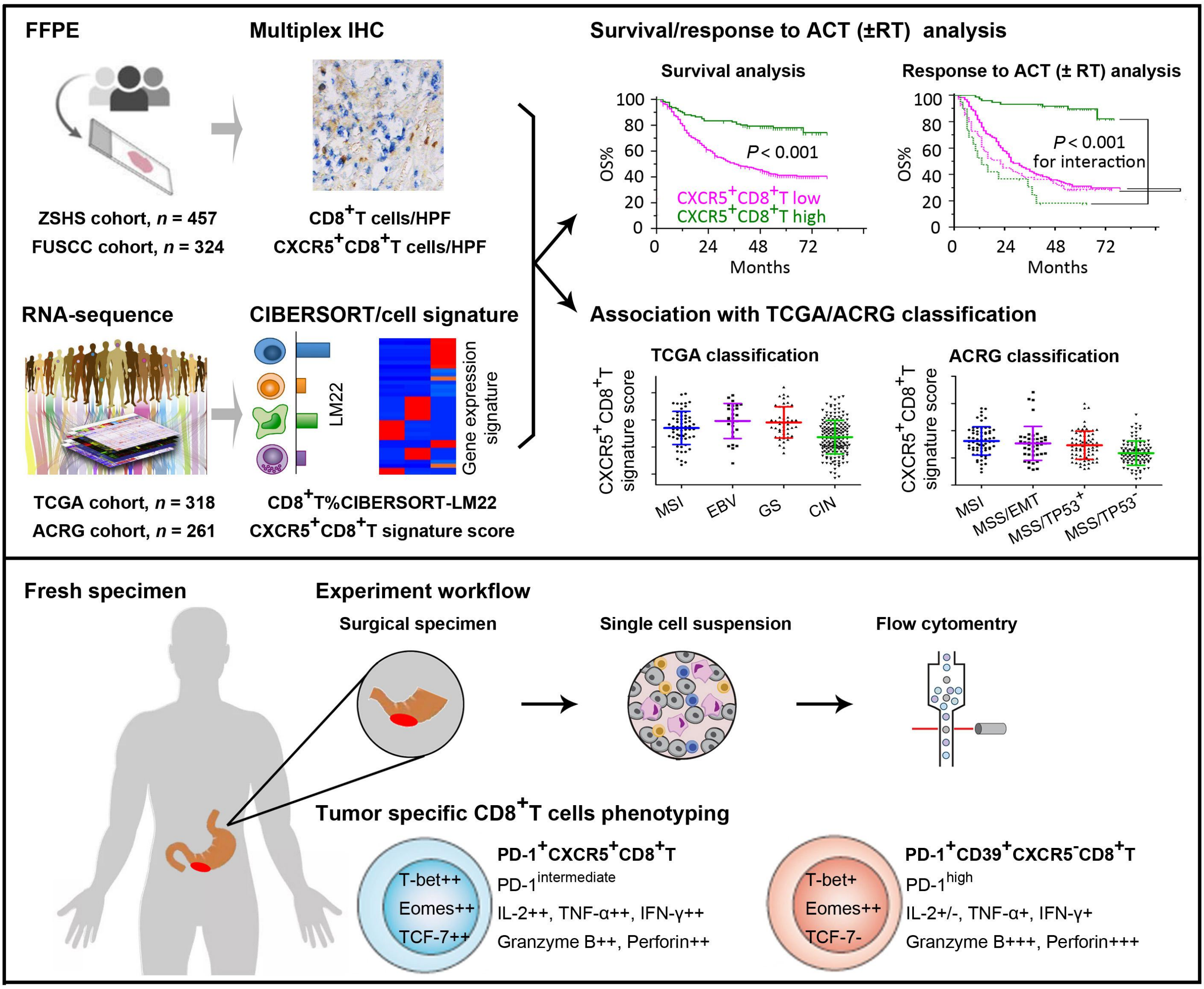

Supplementary Fig. 5 Graphical summary.

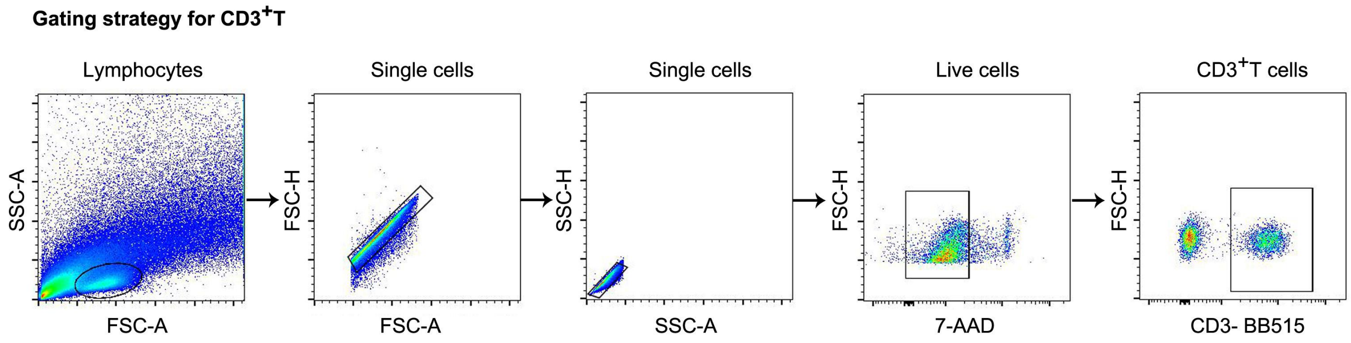

Supplementary Fig. 6 The gating strategy for $CD3^{+}T$ cells in flow cytometry analysis. Gating strategy to determine the $CD3^{+}T$ cells. This gating panel correspond to Fig.1c panel in the manuscript figures.

Supplementary Table 1. Enrolled patient characteristics.

Cohort	ZSHS cohort		FUSCC cohort		TCGA cohort		ACRG cohort	
	No.	%	No.	%	No.	%	No.	%
Total	457	100	324	100	318	100	261	100
Age (year)								
Mean ± SD	60.0±11.7		61.2±10.5		65.0±10.7		61.6±11.5	
Gender								
Male	323	70.7	251	77.5	203	63.8	173	66.3
Female	134	29.3	73	22.5	115	36.2	88	33.7
Histotype								
Adenocarcinoma	382	83.6	256	79.0	296	93.1	210	80.5
Mucinous adenocarcinoma	25	5.5	24	7.4	14	4.4	5	1.9
Signet cell carcinoma	45	9.8	35	10.8	8	2.5	36	13.8
Others	5	1.1	9	2.8	0	0.0	10	3.8
Grade								
G1	14	3.1	2	0.6	5	1.6	9	3.4
G2	94	20.6	82	25.3	106	33.3	97	37.2
G3	349	76.3	240	74.1	207	65.1	155	59.4
Pathological T stage								
T1	78	17.1	9	2.8	15	4.7	0	0.0
T2	65	14.2	10	3.1	64	20.1	156	59.8
T3	90	19.7	28	8.6	152	47.8	86	33.0
T4	224	49	277	85.5	87	27.4	19	7.3
Pathological N stage								
N0	170	37.2	72	22.2	98	30.8	34	13.0
N1	52	11.4	63	19.4	89	28	107	41.0
N2	90	19.7	60	18.5	67	21.1	72	27.6
N3	145	31.7	129	39.8	64	20.1	48	18.4
Metastasis								
Yes	9	2.0	14	4.3	20	6.3	25	9.6
No	448	98.0	310	95.7	298	93.7	236	90.4
TNM stage*								
I	105	23.0	9	2.8	41	12.9	27	10.3
II	107	23.4	71	21.9	102	32.1	76	29.1
III	236	51.6	230	71	143	45	85	32.6
IV	9	2.0	14	4.3	32	10.1	73	28.0
MSI status								
MSI-high	90	19.7	/	/	62	19.5	61	23.4
MSI-low/ MSS	367	80.3	/	/	256	80.5	200	76.6
EBV								
Positive	42	9.2	/	/	27	8.5	18	6.9
Negative	415	90.8	/	/	291	91.5	243	93.1
ACT† (± RT‡)								
Yes	271	59.3	/	/	/	/	126	48.3
No	186	40.7	/	/	/	/	135	51.7
$CD8^+$T cells/ %CIBERSORT-LM22								
High	384	84.0	250	77.2	40	12.6	14	5.4
Low	73	16.0	74	22.8	278	87.4	247	94.6
$CXCR5^+CD8^+$T cells/ signature score								
High	126	27.6	109	33.6	166	52.2	56	21.5
Low	331	72.4	215	66.4	152	47.8	205	78.5

*ZSHS and FUSCC cohorts used AJCC stage 7th, ACRG cohort used AJCC stage 6th, TCGA cohort used AJCC stage 4th/5th/6th/7th; †ACT means adjuvant chemotherapy; ‡RT means adjuvant radiotherapy.

Supplementary Table 2. Association of CXCR5$^+$CD8$^+$T with patient characteristics.

Cohort	ZSHS cohort (n = 457)			FUSCC cohort (n = 324)			TCGA cohort (n = 318)			ACRG cohort (n = 261)		
	CXCR5$^+$CD8$^+$T cells		P value	CXCR5$^+$CD8$^+$T cells		P value	CXCR5$^+$CD8$^+$T		P value	CXCR5$^+$CD8$^+$T		P value
	High	Low		High	Low		High	Low		High	Low	
Total	126	331		109	215		166	152		56	205	
Age (year)			0.014			0.180			0.411			0.382
Mean ± SD	57.8±11.5	60.8±11.6		60.1±10.0	61.7±10.7		65.5±10.5	64.5±11.1		62.8±9.1	61.3±12.0	
Gender			0.211			0.408			0.014			0.549
Male	95	228		81	170		95	108		39	134	
Female	31	103		28	45		71	44		17	71	
Histotype			0.414			0.654			0.129			0.015
Adeno	105	277		82	174		159	137		48	162	
Mucinous	7	18		10	14		4	10		1	4	
Signet cell	11	34		14	21		3	5		2	34	
Others	3	2		3	6		0	0		5	5	
Grade			0.087			0.178			<0.001			0.007
G1	5	9		0	2		2	3		0	9	
G2	22	72		22	60		36	70		13	84	
G3	99	250		87	153		128	79		43	112	
Pathological T stage			0.026			0.445			0.259			0.062
T1	21	57		2	7		5	10		0	0	
T2	28	37		2	8		36	28		41	115	
T3	22	68		7	21		75	77		13	73	
T4	55	169		98	179		50	37		2	17	
Pathological N stage			0.104			0.399			0.663			0.018
N0	53	117		20	52		51	47		13	21	
N1	19	33		22	41		51	38		17	90	
N2	23	67		25	35		32	35		19	53	
N3	31	114		42	87		32	32		7	41	
Metastasis			0.460			0.063			0.370			0.745
Yes	1	8		1	13		8	12		6	19	
No	125	323		108	202		158	140		50	186	
TNM stage*			0.009			0.050			0.650			0.014
I	32	73		1	8		20	21		12	15	
II	41	66		22	49		53	49		14	62	
III	52	184		85	145		79	64		19	66	
IV	1	8		1	13		14	18		11	62	
MSI status			0.076						0.061			<0.001
MSI-high	31	59		/	/		39	23		23	38	
MSI-low/ MSS	95	272		/	/		127	129		33	167	
EBV			0.058						0.006			<0.001
Positive	15	27		/	/		21	6		15	3	
Negative	111	304		/	/		145	146		41	202	
ACT† (± RT‡)			0.420									0.372
Yes	79	192		/	/		/	/		30	96	
No	47	139		/	/		/	/		26	109	

*ZSHS and FUSCC cohorts used AJCC stage 7th, ACRG cohort used AJCC stage 6th, TCGA cohort used AJCC stage 4th/5th/6th/7th; †ACT mean[illegible] adjuvant chemotherapy; ‡RT means adjuvant radiotherapy; P value shown resulted from the association between CXCR5$^+$CD8$^+$T and clinica[illegible] factors, using student's t test (two-sided, continuous variables), Chi-squared test (categorical variables) and Fisher's exact test (categorical variable[illegible] violating the rules of Chi-squared test).

Supplementary Table 3. Association of CXCR5$^+$CD8$^+$T with patient characteristics in the ZSHS and ACRG TNM II+III cohorts.

Cohort	ZSHS TNM II+III cohort (n = 343)			ACRG TNM II+III cohort (n = 161)		
	CXCR5$^+$CD8$^+$T cells		P value	CXCR5$^+$CD8$^+$T signature score		P value
	High	Low		High	Low	
Total	93	250		33	128	
Age			0.033			0.239
Mean ± SD	58.3±11.6	61.3±11.5		64.2±8.5	61.6±11.4	
Gender			0.494			0.656
Male	70	177		25	92	
Female	23	73		8	36	
Histotype			0.388			0.023
Adenocarcinoma	77	211		29	107	
Mucinous adenocarcinoma	7	17		1	2	
Signet cell carcinoma	6	20		0	17	
Others	3	2		3	2	
Grade			0.334			0.080
G1	0	0		0	8	
G2	13	48		9	52	
G3	80	202		24	68	
Pathological T stage			0.098			0.878
T1	1	4		0	0	
T2	15	18		22	85	
T3	22	64		11	42	
T4	55	164		0	1	
Pathological N stage			0.178			0.495
N0	23	48		1	4	
N1	16	28		17	80	
N2	23	63		15	44	
N3	31	111		0	0	
TNM stage*			0.003			0.539
II	41	66		14	62	
III	52	184		19	66	
MSI status			0.272			0.172
MSI-high	20	41		11	28	
MSI-low/ MSS	73	209		22	100	
EBV			0.581			<0.001
Positive	10	22		10	2	
Negative	83	228		23	126	
ACT┼ (± RT╪)			0.176			0.419
Yes	74	179		21	69	
No	19	71		12	59	

*ZSHS cohort used AJCC stage 7th, ACRG cohort used AJCC stage 6th; ┼ACT means adjuvant chemotherapy; ╪RT means adjuvant radiotherapy; P value shown resulted from the association between CXCR5$^+$CD8$^+$T and clinical factors, using student's t test (two-sided, continuous variables), Chi-squared test (categorical variables) and Fisher's exact test (categorical variables violating the rules of Chi-squared test).

Supplementary Table 4. Multivariate Cox analyses in the ZSHS TNM II+III cohort (n = 343).

Patients characteristic	$CD8^+T$ cells low	$CD8^+T$ cells high	$CXCR5^+CD8^+T$ cells low	$CXCR5^+CD8^+T$ cells high
	HR (95%CI)	HR (95%CI)	HR (95%CI)	HR (95%CI)
Total	56	287	250	93
ACT†				
Yes vs. no	0.1640 (0.0551-0.4884)	0.3489 (0.2404-0.5063)	0.4875 (0.3347-0.7100)	0.0575 (0.0159-0.2080)
Age (years)	0.9952 (0.9554-1.0367)	1.0051 (0.9905-1.0198)	0.9973 (0.9836-1.0111)	1.0391 (0.9826-1.0990)
Gender				
Male vs. female	0.9241 (0.4202-2.0321)	0.8787 (0.6191-1.2474)	1.0324 (0.7326-1.4549)	0.3505 (0.1144-1.0735)
Histotype				
Mucinous vs. adeno	0.7809 (0.2622-2.3252)	0.8969 (0.4513-1.7823)	0.8843 (0.4691-1.6669)	0.4259 (0.1011-1.7946)
Signet cell vs. adeno	1.8799 (0.6676-5.2940)	1.8175 (0.9612-3.4366)	1.8823 (1.0770-3.2899)	1.8771 (0.2032-17.3397)
Others vs. adeno	1.2017 (0.1464-9.8610)	0.0 ($0.0-7.85*10^{236}$)	0.2200 (0.0301-1.6059)	0.0 ($0.0-1.01*10^{304}$)
Grade				
G3 vs. G2	1.0372 (0.2572-4.1822)	0.8640 (0.5409-1.3799)	0.7524 (0.4752-1.1913)	7.6458 (0.7744-75.4876)
Pathological T stage				
T2 vs. T1	/	0.8161 (0.1757-3.7908)	2.0414 (0.2595-16.0589)	0.0990 (0.0046-2.1170)
T3 vs. T1 (T3 vs. T2)	4.7606 (0.8737-25.9391)	1.6188 (0.3788-6.9185)	3.3444 (0.4538-24.6503)	0.1746 (0.0111-2.7352)
T4 vs. T1 (T4 vs. T2)	5.2049 (0.9769-27.7305)	1.6024 (0.3827-6.7105)	4.0220 (0.5514-29.3347)	0.1281 (0.0080-2.0393)
Pathological N stage				
N1 vs. N0	0.2174 (0.0373-1.2676)	2.5074 (1.2939-4.8592)	2.3473 (1.2313-4.4751)	0.8263 (0.0582-11.7392)
N2 vs. N0	0.4212 (0.0827-2.1454)	2.3127 (1.2999-4.1148)	1.9122 (1.0846-3.3716)	2.6732 (0.5072-14.0895)
N3 vs. N0	1.1555 (0.2852-4.6821)	4.7890 (2.8001-8.1907)	4.1524 (2.4225-7.1178)	4.6349 (1.0696-20.0847)
MSI				
MSI-high vs. MSI-low/ MSS	0.2323 (0.0632-0.8535)	0.8144 (0.5318-1.2473)	0.7540 (0.4914-1.1569)	0.6451 (0.1415-2.9416)
EBV status				
Positive vs. negative	0.7095 (0.1588-3.1698)	0.3573 (0.1653-0.7719)	0.5034 (0.2602-0.9738)	0.0 ($0.0-2.46*10^{251}$)

ZSHS cohort used AJCC stage 7th; †ACT means adjuvant chemotherapy.

Supplementary Table 5. Multivariate Cox analyses in the ACRG TNM II+III cohort (*n* = 161).

Patients characteristic	CD8$^+$T low	CD8$^+$T high$^{\#}$	CXCR5$^+$CD8$^+$T low	CXCR5$^+$CD8$^+$T high
	HR (95%CI)	HR (95%CI)	HR (95%CI)	HR (95%CI)
Total	156	5	128	33
ACT† ± RT‡				
Yes vs. no	0.4198 (0.2500-0.7049)	/	0.4246 (0.2428-0.7426)	0.0052 (0.0001-0.4292)
Age (years)	1.0180 (0.9918-1.0447)	/	1.0207 (0.9940-1.0481)	1.0258 (0.9129-1.1526)
Gender				
Male vs. female	1.4638 (0.7753-2.7638)	/	1.9077 (0.9796-3.7151)	0.0220 (0.0009-0.5657)
Histotype				
Mucinous vs. adeno	0.0 (0.0-7.56*10^{209})	/	0.0 (0.0-6.66*10^{233})	0.0 (0.0-1.01*10^{304})
Signet cell vs. adeno	1.1333 (0.4808-2.6710)	/	1.0226 (0.4336-2.4116)	/
Others vs. adeno	0.4021 (0.0541-2.9888)	/	1.5683 (0.2004-12.2762)	0.0001 (0.0-2.59*10^{244})
Grade				
G2 vs. G1	1.3957 (0.4086-4.7676)	/	1.4547 (0.4219-5.0153)	/
G3 vs. G1 (G3 vs. G2)	1.4392 (0.4172-4.9642)	/	1.7941 (0.5143-6.2586)	0.0364 (0.0016-0.8210)
Pathological T stage				
T3 vs. T2	1.4735 (0.8598-2.5250)	/	1.5693 (0.8882-2.7729)	9.3784 (0.6244-140.8634)
T4 vs. T2	0.0 (0.0-1.01*10^{304})	/	0.0 (0.0-1.01*10^{304})	/
Pathological N stage				
N1 vs. N0	4.0706 (0.5354-30.9480)	/	2.9243 (0.3728-22.9400)	1.24*10^{7} (0.0-1.01*10^{304})
N2 vs. N0	7.7900 (1.0259-59.1510)	/	6.9313 (0.8646-55.5677)	2.88*10^{5} (0.0-1.01*10^{304})
MSI				
MSI-high vs. MSI-low/ MSS	0.4322 (0.2265-0.8247)	/	0.4383 (0.2131-0.9015)	0.8642 (0.1069-6.9840)
EBV status				
Positive vs. negative	0.8317 (0.3223-2.1462)	/	1.1240 (0.1417-8.9137)	0.8527 (0.1280-5.6818)

ACRG cohort used AJCC stage 6th; †ACT means adjuvant chemotherapy; ‡RT means adjuvant radiotherapy. $^{\#}$Sample size too small to conduct multivariate Cox analysis.

Supplementary Table 6. Antibodies.

Name	Company	Clone	Catalogue No.	Application	Diluted
BV421 Mouse Anti-Human CD45	BD Biosciences	HI30	563879	FC†	1/20
FITC Mouse Anti-Human CD3	BD Biosciences	HIT3a	555339	FC	1/20
Alexa Fluor® 700 anti-human CD8a Antibody	BioLegend	RPA-T8	301028	FC	1/20
BV421 Rat Anti-Human CXCR5 (CD185)	BD Biosciences	RF8B2	562747	FC	1/20
PE anti-human CD279 (PD-1) Antibody	BioLegend	EH12.2H7	329906	FC	1/20
BV786 Mouse Anti-Human CD152	BD Biosciences	BNI3	563931	FC	1/20
Alexa Fluor® 647 Mouse Anti-Human TIM-3 (CD366)	BD Biosciences	7D3	565558	FC	1/20
Brilliant Violet 785™ anti-human CD223 (LAG-3) Antibody	BioLegend	11C3C65	369322	FC	1/20
PE anti-human TIGIT (VSTM3) Antibody	BioLegend	A15153G	372704	FC	1/20
Alexa Fluor® 647 Mouse Anti-Human CD107A	BD Biosciences	H4A3	562622	FC	1/20
PE Mouse Anti-Human Granzyme B	BD Biosciences	GB11	561142	FC	1/20
Alexa Fluor® 647 Mouse Anti-Human Perforin	BD Biosciences	δG9	563576	FC	1/20
PE Rat Anti-Human IL-2	BD Biosciences	MQ1-17H12	559334	FC	1/20
Alexa Fluor® 647 anti-human TNF-α Antibody	BioLegend	MAb11	502916	FC	1/20
BV786 Mouse Anti-Human IFN-γ	BD Biosciences	4S.B3	563731	FC	1/20
PE-Cy™7 Mouse anti-Ki-67	BD Biosciences	B56	561283	FC	1/20
PE anti-mouse CD103 Antibody	BioLegend	2E7	121406	FC	1/20
Alexa Fluor® 647 Rat Anti-Human CCR7 (CD197)	BD Biosciences	3D12	557734	FC	1/20
BV786 Mouse Anti-Human CD69	BD Biosciences	FN50	563834	FC	1/20
PE Mouse Anti-Human CD62L	BD Biosciences	DREG-56	555544	FC	1/20
PE-Cy™7 Mouse Anti-Human CD127	BD Biosciences	HIL-7R-M21	560822	FC	1/20
Alexa Fluor® 647 anti-TCF1 (TCF7) Antibody	BioLegend	7F11A10	655204	FC	1/20
Alexa Fluor® 647 anti-human CD27 Antibody	BioLegend	M-T271	356434	FC	1/20
EOMES Monoclonal Antibody (WD1928), PE, eBioscience™	ThermoFisher Scientific	WD1928	12-4877-42	FC	1/20
BV786 Mouse Anti-T-bet	BD Biosciences	O4-46	564141	FC	1/20
Alexa Fluor® 647 Mouse anti-Bcl-6	BD Biosciences	K112-91	561525	FC	1/20
CXCL13 Monoclonal Antibody (53610), PE	ThermoFisher Scientific	53610	MA5-23666	FC	1/20
7-AAD	BD Biosciences	-	559925	FC	1/20
PE/Cy7 Annexin V	BioLegend	-	640950	FC	1/20
CD39 Monoclonal Antibody (eBioA1 (A1)), FITC, eBioscience™	ThermoFisher Scientific	eBioA1 (A1)	11-0399-42	FC	1/20
PerCP/Cyanine5.5 anti-human CD279 (PD-1) Antibody	BioLegend	A17188B	621614	FC	1/20
Brilliant Violet 605™ anti-human CD152 (CTLA-4) Antibody	BioLegend	BNI3	369610	FC	1/20
Brilliant Violet 650™ anti-human TNF-α Antibody	BioLegend	MAb11	502938	FC	1/20
Alexa Fluor® 647 Mouse anti-Human Granzyme B	BD Biosciences	GB11	560212	FC	1/20
PE anti-human Perforin Antibody	BioLegend	B-D48	353304	FC	1/20
BV605 Rat Anti-Human IL-2	BD Biosciences	MQ1-17H12	564165	FC	1/20
TOX Monoclonal Antibody (TXRX10), PE, eBioscience™	ThermoFisher Scientific	TXRX10	12-6502-82	FC	1/20
Anti-CD8 alpha antibody [C8/468 + C8/144B]	Abcam	C8/468+C8/144B	ab199016	IF‡	1/200
Recombinant Anti-CXCR5 antibody [EPR23463-30]	Abcam	EPR23463-30	ab254415	IF	1/5000
Mouse Anti-CD8 alpha antibody	Abcam	144B	ab17147	IHC*	1/50
Anti-CXCR5 antibody	Abcam	Polyclonal	ab46218	IHC	1/1000

†FC means flow cytometry; ‡IF means immunoflourescence; *IHC means immunohistochemistry.
